# A customized convolutional neural network-based approach for weeds identification in cotton crops

**DOI:** 10.3389/fpls.2024.1435301

**Published:** 2025-01-08

**Authors:** Hafiz Muhammad Faisal, Muhammad Aqib, Khalid Mahmood, Mejdl Safran, Sultan Alfarhood, Imran Ashraf

**Affiliations:** ^1^ University Institute of Information Technology (UIIT), Pir Mehr Ali Shah (PMAS)-Arid Agriculture University, Rawalpindi, Pakistan; ^2^ National Center of Industrial Biotechnology, Pir Mehr Ali Shah (PMAS)-Arid Agriculture University Rawalpindi, Punjab, Pakistan; ^3^ Institute of Computing and Information Technology, Gomal University, D.I. Khan, Pakistan; ^4^ Department of Computer Science, College of Computer and Information Sciences, King Saud University, Riyadh, Saudi Arabia; ^5^ Information and Communication Engineering, Yeungnam University, Gyeongsan, Republic of Korea

**Keywords:** deep learning, convolutional neural networks, object classification, cotton crops weeds, weeds detection

## Abstract

Smart farming is a hot research area for experts globally to fulfill the soaring demand for food. Automated approaches, based on convolutional neural networks (CNN), for crop disease identification, weed classification, and monitoring have substantially helped increase crop yields. Plant diseases and pests are posing a significant danger to the health of plants, thus causing a reduction in crop production. The cotton crop, is a major cash crop in Asian and African countries and is affected by different types of weeds leading to reduced yield. Weeds infestation starts with the germination of the crop, due to which diseases also invade the field. Therefore, proper monitoring of the cotton crop throughout the entire phases of crop development from sewing to ripening and reaping is extremely significant to identify the harmful and undesired weeds timely and efficiently so that proper measures can be taken to eradicate them. Most of the weeds and pests attack cotton plants at different stages of growth. Therefore, timely identification and classification of such weeds on virtue of their symptoms, apparent similarities, and effects can reduce the risk of yield loss. Weeds and pest infestation can be controlled through advanced digital gadgets like sensors and cameras which can provide a bulk of data to work with. Yet efficient management of this extraordinarily bulging agriculture data is a cardinal challenge for deep learning techniques too. In the given study, an approach based on deep CNN-based architecture is presented. This work covers identifying and classifying the cotton weeds efficiently alongside a comparison of other already existing CNN models like VGG-16, ResNet, DenseNet, and Xception Model. Experimental results indicate the accuracy of VGG-16, ResNet-101, DenseNet-121, XceptionNet as 95.4%, 97.1%, 96.9% and 96.1%, respectively. The proposed model achieved an accuracy of 98.3% outperforming other models.

## 1 Introduction and literature review

Smart farming is revolutionized by the use of the Internet of Things (IoT) and artificial intelligence (AI) Imran et al. (2018); Guo et al. (2020). The use of smart technology, especially sensors, and IoT, has significantly increased in smart farming Jayaraman et al. (2016). Sensors deployed in agricultural fields generate huge amounts of data on a daily basis, which could be named agricultural big data. Based on this data, diseases, and weeds could be detected at a premature stage by applying various computer vision and deep learning techniques. This will not only benefit farmers but could also help deal with the issue of shortage of crop production globally. An estimated 20 billion is lost worldwide just because of low crop yields due to different reasons including weeds. A controlling system, such as sprayers for precisely spraying unwanted objects, can be developed using smart technology to manage weeds. Such systems can increase yield and can also reduce production costs and labor Escalante et al. (2019).

Precise weed management in crops is one of the biggest challenges that could be handled using precision agriculture techniques. Diseases in plants and leaves are directly proportional to the yield of any crop, and most of the plant diseases are caused by weeds Capinera (2005); Kumar et al. (2021). Plant production can easily be increased if weeds are destroyed in time. The most difficult thing for researchers to do is to identify multiple types of weeds in different environmental conditions. Traditional methods for the detection of different weeds are expensive and time-consuming. Therefore, there is a need for an approach that can quickly identify the weeds within a short amount of time. Deep learning, computer vision, and machine learning (ML) advancements in recent years have the potential to alter and modernize how crops are grown, managed, and harvested. In deep learning, features are automatically extracted, which gives it an advantage over machine learning Dokic et al. (2020). Weeds are dangerous for crops and plants as they consume resources such as stealing of water, nutrients as well as sunshine causing low-quality yield. With ground-breaking research in computer vision, state-of-the-art algorithms have the potential to be applied in effective crop yield prediction.

Deep learning has many techniques like classic neural networks, convolutional neural networks (CNN), recurrent neural networks (RNN), generative adversarial networks (GAN), self-organizing maps, Boltzmann machines, and many more (Grigorescu et al., 2020). From cotton crop cultivation to harvest, it takes about four months. As soon as the crop is planted, the weeds begin to grow, and these weeds cause disease in the cotton crop. Most weeds are similar in shape. It is a difficult step to detect, classify, and then destroy such weeds in time. This study aims at designing an efficient model to accurately classify cotton weeds. The following are the key contributions of this research.

Nowadays, the use of unmanned aerial vehicles (UAVs) has revolutionized agriculture. UAVs are not only used for data collection and uniform spraying of agrochemicals but they are now used for precise weeds management as well by detecting weeds and precisely spraying agrochemicals on them (Khan et al., 2021; Olsen et al., 2019). This not only helps to reduce to quantity of weedicides but also saves money, and time and increases agricultural production. Some crops like cotton need care on a daily basis and UAVs could be very effective in the timely detection of weeds and thus they could be sprayed properly. UAV-based automated spraying systems use deep learning (DL) techniques for the detection and classification of weeds in an efficient manner.

- Real field weeds data acquisition from cotton crops under various climatic and illumination conditions.
- Proposed a CNN-based deep learning approach for weed detection and classification. Performance analysis of the models concerning accuracy and loss and k-fold cross-validation.
- Analyze and compare the results of the proposed deep learning-based approach to prior existing approaches to see the potential of the proposed model.
- To collect cotton crop data of six different weeds from a real environment. Weeds include Wild Cucurbit, Slender Amaranth, Nut Grass, Horse Purslane, Common Puncture Vine, and Trefoil.
- To employ deep learning techniques in a manner that would enable them to categorize data based on shared illness signs in cotton crops.

An overview of recent relevant works that use computer vision and DL for weed detection and classification is given here. Literature also describes a variety of datasets and multiple deep learning algorithms for the classification of different species of weeds, under different environmental conditions.

An AI-based model for weeds classification and diseases in crops was proposed by Saiz-Rubio and Rovira-Más (2020) in the area of smart farming. UAVs were used for harvesting, irrigation, weed detection, disease detection, seedlings, and spraying. A smart decision support system (SDSS) was used for real-time analysis using G5 technology, especially for irrigation, and also improved water and land efficiency. The transfer learning technique of DL was used with the help of the DenseNet for recognition of the growth stage of weeds. A publicly available dataset was used containing 18 classes of weeds. The result of the proposed model has been compared with ResNet, MobileNet, Wide-ResNet, and DenseNet, and the proposed model achieved 93.45% accuracy (Vypirailenko et al., 2021).

You only look once (YOLOv3) algorithm, PyTorch, and Keras frameworks were used for the classification of common weeds in corn and soybean crops. The dataset contains only 462 images, which were collected from publicly available dataset (Dataset Weed Images, 2022). The size of the dataset was very small. They have achieved good accuracy of up to 98.8% by applying the VGG-16. While they have given good results, there can be a tendency for lower graph accuracy with a large dataset (Ahmad et al., 2021).

Luo et al. (2023) used a CNN model for weed classification. The dataset consisted of 140 species of weed seeds, which were collected from a forest in China, and classified manually by an expert. 14096 images were used for testing purposes and 33600 images were used for training the model. Six different CNN models i.e. AlexNet, NasNet, VGG-16, SqueezeNet, Xception, and GoogleNet have been used. GoogleNet achieved the highest results. Another group of researchers carried out semantic segmentation for weed detection from canola crop fields with the help of a deep neural network (Asad and Bais, 2020). The dataset was collected from Manitoba Canada, which contains only 906 images belonging to two classes. Results were compared with UNET-VGG16, UNET-ResNet50, SegNet-VGG16, and SegNet-ResNet50. The deployed semantic segmentation approach showed an accuracy of 98.23% with a 99.2% F1 score. However, this model can be improved by using an enriched dataset with multiple species of weeds and more images.

Grace et al. (2021) identified crops and weeds, using the CNN model of deep learning for this purpose. The dataset was collected from Kaggle, and the size of the dataset was very small, it contains only 960 images. The dataset for training and testing was split into 80:20 ratios respectively. All the experiments are performed using Google Colaboratory. The resulting accuracy of the proposed algorithm was 89%. Therefore, the proposed approach proves to be better than AlexNet.

Dadashzadeh et al. (2020) proposed a stereo vision system for weed and rice by implementing PSO and bee algorithm has been used. The dataset was in the form of stereo videos, which were collected from rice fields then it was analyzed with the help of MATLAB. Results were compared with K nearest neighbor (KNN) classifier, and the proposed classifier performed better as compared to KNN. Geometric mean and arithmetic mean were used as performance metrics.

Classification of herbs in the field of turfgrass was done through VGG CNN (Yu et al., 2019). The dataset was calculated from different grassy grounds in America. 36,000 images, 18,000 each for positive and negative classes. Result of VGGNet compared with GoogleNet, the performance of VGGNet was better as compared to GoogleNet. Weed recognition using DL and image processing using genetic algorithm, and CenterNet model is carried out by (Jin et al., 2021). A dataset of white cabbage vegetable plants was collected from vegetable fields in China, a total of 1150 images were used for training purposes, and the size of the dataset was very small. The result of the proposed CenterNet model was an F1-score of 0.953, precision of 95.6%, and recall of 95.0%.

Olsen et al. (2019) used Inception V3 and ResNet50 DL models for weed classification with the help of a robot. The dataset consists of 17,509 images, which were collected from North Australian fields. ResNet50 and Inception V3 achieved average performance accuracy of 97.6% and 95.1%, respectively. Sensors were used for the classification of weeds and carrot plants with the help of CNN models and the TensorFlow framework. The dataset consisted of 36000 carrot plants and 36000 images of weed plants. The result of the proposed model according to performance metrics was 96.41%, 98.9%, 96.82%, and 97.59%, respectively (Knoll et al., 2019).

ML played an important role in implementing different precision agriculture techniques. Benos et al. (2021) used ML algorithms like SVM and BPNN are used for the detection of weeds. Both algorithms have achieved better performance, overall accuracy of 95.069 percent and 96.70 percent are achieved for SVM and BPNN respectively (Abouzahir et al., 2018). But, further improvement could be made in performance by using a variety of datasets, collected under different lighting conditions, collecting data of different varieties of crops, etc. Machine learning has several limitations in terms of higher error, time consumption, algorithm selection, and feature extraction problems (Dokic et al., 2020).

Ruslan et al. (2022) used ML and image processing techniques for the classification of the weedy seed of rice with the help of different seven classifiers. For coloring purposes, three types of parameters were used color, texture, and morphology to enhance the performance. The total sample of weedy seed images was 7350. Performance was measured with sensitivity, specificity, accuracy, and average correct classifier, the output of these performance metrics were 85.3%, 99.5%, 97.9%, and 92.4% respectively. Similarly, Espinoza (2020) carried out weed detection with a focus on a real-time analysis performed after collecting the dataset and using it to train algorithms such as YOLO, Faster R-CNN, and a mobile algorithm i.e. single shot detection (SSD). A UAV was deployed on the fields to collect images of strawberry plants as well as weeds to build a dataset for training deep learning architectures. A key issue for weed detection is the similar structure and shape of both plants and weeds making it quite hard to recognize between the plants and their weeds.

Valicharla (2021) worked on weed classification and detection using DL algorithms. For this purpose, they have used the Mask R-CNN model with the help of pixel-wise segmentation. In this work, they have used a synthetic dataset of 200 images collected from a carrot field. Loss and accuracy results obtained during model training have been compared by implementing the VGG-19 model. The highest accuracy reported by the proposed model is 92%. Although they have achieved good results, there could be a decreasing trend in the accuracy graph by increasing the dataset size and adding a variety of images to it.

The literature discussed above has shown good performance using deep learning CNN models to classify and detect weeds. However, weed detection and classification is still a challenging task and comes with many limitations such as a small dataset, and less number of weed species. In addition, low-quality images from controlled environments can greatly affect the accuracy. The objective of this research is to develop a DL-based CNN model for weed detection and classification under different environmental conditions in a timely manner to eradicate weeds in cotton crops.

Further, the proposed methodology for weeds detection and classification is presented in Section 2. All the details of experimentation and their results are discussed in Section 3. Lastly, the conclusion is given in Section 4.

## 2 Proposed methodology

**Figure 1:**
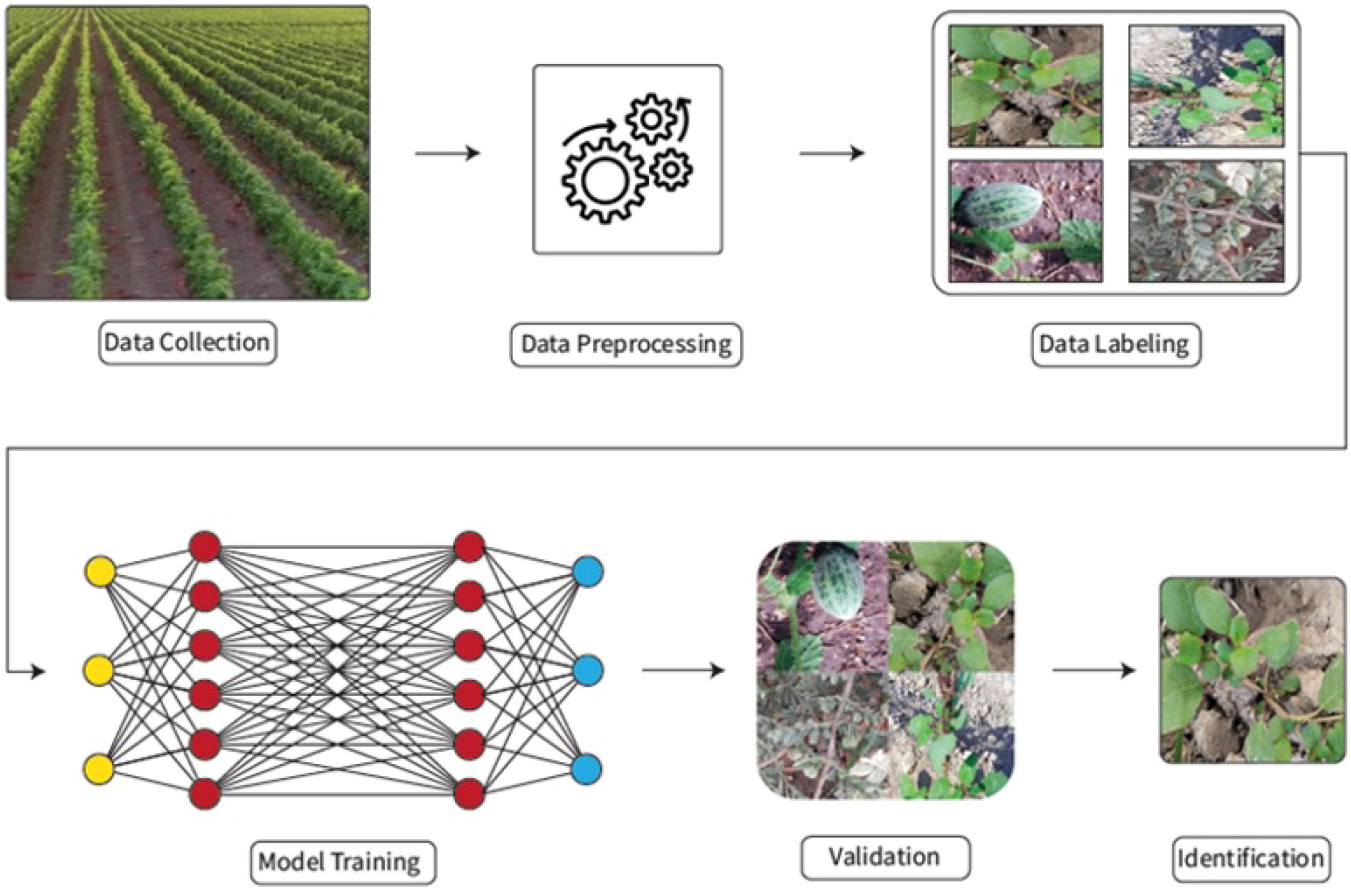
Weeds detection model workflow.

In this section, an overview of the DL-based weed detection and classification methodology is given and a detailed description of the detection workflow is presented in **Figure 1**. The initial step of the methodology is the data collection i.e. collection of data from the field, which is then processed using pre-processing techniques. To overcome over-fitting issues, different data augmentation techniques are applied and afterward, the dataset is properly annotated and labeled before using it as input for model training. For model training, CNN-based models are trained using the input dataset and trained models are then used for the prediction and classification of weeds after evaluating the prediction accuracy of these models.

### 2.1 Data collection and preprocessing

In this section, all the details regarding data collection from the field and then its preprocessing are discussed. Images of different kinds of weeds found in the cotton crop during the summer season are collected. The data was collected from an irrigated cotton field in Rahim Yar Khan, a city in the Southern Punjab region of Pakistan. The area of the selected field is 12 acres and only the cotton crop is grown in this field. Data for this purpose is collected through two different mobile devices (vivo 1920 and iPhone 6S), where the resolution of both the cameras in those devices is 48 megapixels with an aperture of f/2.2, 13mm (ultra-wide).

The data is collected at four different time intervals of the day, from sunrise in the morning to sunset in the evening. The data is collected in the month of August and during this month, the sun rises around 5:35 am and sets around 6:40 pm in the selected region of south Punjab. So, the first interval starts early morning before sunrise from 5:30 am to 7:00 am. Then after a break of 2 hours, data is collected around the midday time starting from 9:00 am to 11:00 am. The third interval starts after noon from 12:30 pm to 2:00 pm and the fourth interval starts in the evening from 5:00 pm to 7:00 pm. Dataset collected in this work is freely available and can be accessed using DOI 10.5281/zenodo.8383873 and https://doi.org/10.34740/KAGGLE/DS/3095815. In the month of August, the weather of South Punjab remains very hot and dry and the temperature in a day remains between 88° F to 100° F and sometimes goes beyond the upper limit. Humidity is always high during this period and remains between 40% to 50%. During normal weather conditions, more than 14000 images are captured in.JPG format with a resolution of 1280×720.

In order to collect data on weeds that grow in different crop age periods, the crop was monitored from germination to production. The age of the cotton crop is about four months, and the growth of the weeds starts right from the beginning. In this work, the data of six different types of weeds is collected and each type of weed has more than 2000 images.

**Figure 2:**
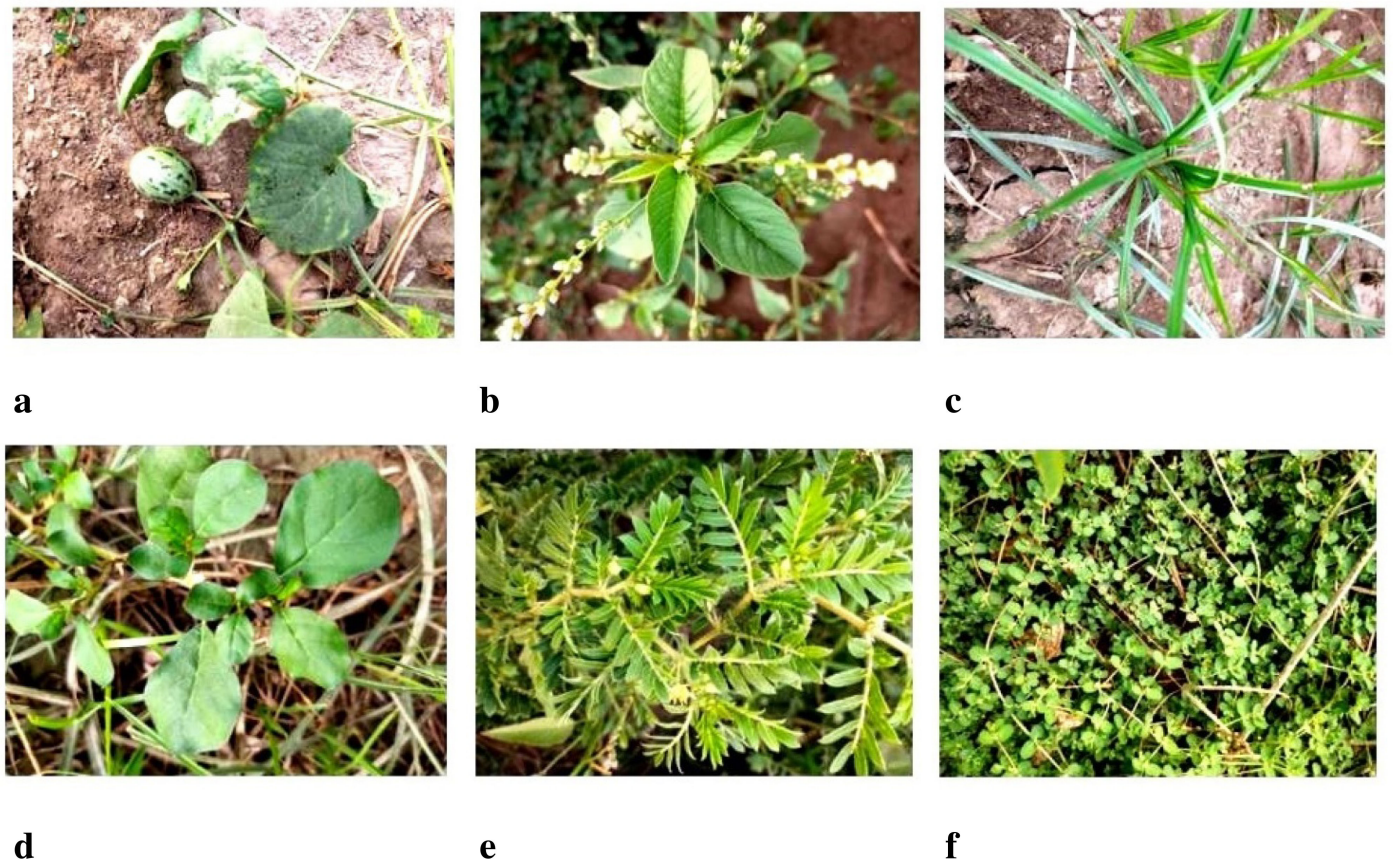
Classes of weeds collected in the dataset, **(A)** Wild cucurbit, **(B)** Slender amaranth, **(C)** Nut grass, **(D)** Horse purslane, **(E)** Common puncture vine, and **(F)** Trefoil.

In **Figure 2**, all six types of weeds i.e. ‘Wild Cucurbit’, ‘Slender Amaranth’, ‘Nut Grass’, ‘Horse Purslane’,’Common Puncture Vine’ and ‘Trefoil’ Xu and Chang (2017) are shown where the weed shown in **Figure 2A** is the Wild Cucurbit. This weed is in the shape of a vine, and it also appears as soon as the cotton plant emerges from the ground. Wild Cucurbit seed is naturally hidden in the ground. The vine of the wild cucurbit grips the cotton plant, which stops the growth of the cotton plant, and the vine produces a stalk, which destroys the tiny leaves and buds of the cotton and leads to the death of the cotton plant.

In **Figure 2B**, Slender Amaranth is shown and the leaves of this weed are somehow similar to those of cotton leaves at the time of germination. Pest is also produced on this weed, which affects the cotton crop. In **Figure 2C**, Nut Grass is depicted as a weed that causes disease in cotton crops, not only damaging the plants but also inhibiting their growth. In **Figure 2D**, ‘Horse Purslane’ is shown which is considered very dangerous for the crop. Its growth starts with the growth of the cotton crop and it spreads very fast. Due to this weed, pests attack the crop and if it is not controlled in time, the cotton crop is destroyed. In addition to the pest attack, this weed also spreads many diseases.

In **Figure 2E**, ‘Common Puncture Vine’ is shown which is not only dangerous for the cotton crop but also harmful for human health. It is a vine-shaped weed with triangular thorns which are also called the seeds of this weed. Due to this, it make it difficult for farmers to move in the cotton field because a painful sting is produced on this weed. Pests are also produced on this weed which affects the cotton crop and production. In **Figure 2F**, ‘Laiti Vine Soft’ is shown which spreads on the ground in the form of vines and produces pests that can damage the cotton crop as well.

### 2.2 Data preprocessing

After data collection, the next phase is data preprocessing. Before inputting the images into the model, several preprocessing steps are typically employed to enhance the quality of images and extract relevant information from the images. First, image normalization is performed to ensure consistent lighting conditions across the dataset, which involves adjusting brightness, contrast, and color balance. Next, image resizing is carried out to standardize the input dimensions, reducing computational complexity while maintaining essential details.

Augmentation techniques are one of the common ways to capture more patterns in the dataset by a number of techniques such as rotation, zooming, flipping, brightness enhancement, and contrast adjustment, to name a few. These techniques result in new images that can be exposed (given) to the deep learning model while training to improve its detection accuracy and robustness.

**Figure 3:**
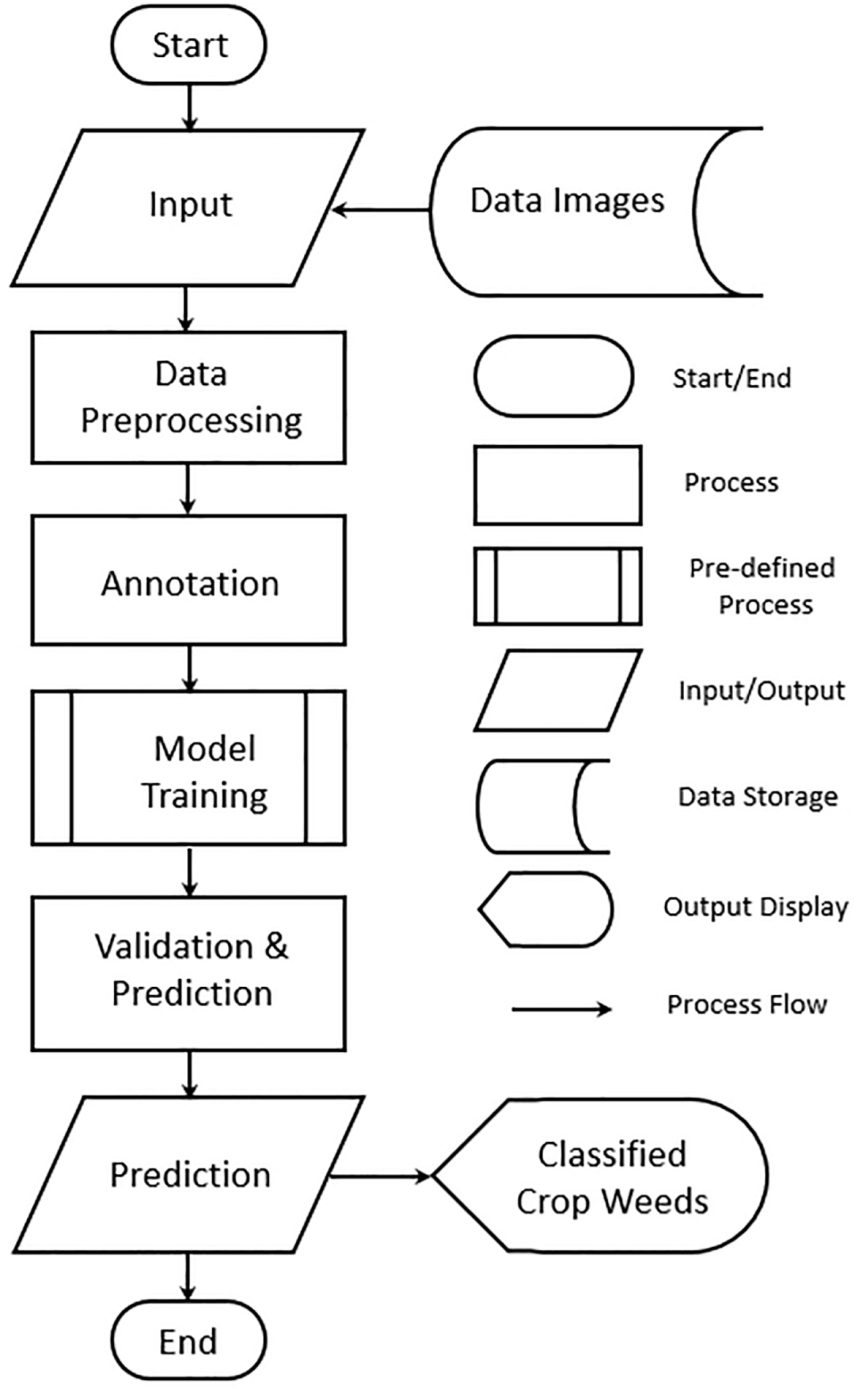
Flowchart of model training.

**Figure 3** shows the workflow of the proposed approach. The workflow initiates at the “Start” point, marking the beginning of the weed classification process. The system starts by receiving input in the form of data images, which are images of crops that potentially contain weeds. These images form the foundational dataset for training and validating the model. Capturing diverse images that represent different weed types, growth stages, and environmental conditions is essential to improve the robustness of the classification model. In this stage, raw images have image processing to enhance their quality and ensure consistency in the dataset. Common preprocessing tasks may include resizing (to standardize dimensions), normalization (to scale pixel values), and augmentation (to generate variations by flipping, rotating, or adjusting brightness). The goal of preprocessing is to optimize the images for model training and to create a dataset that allows the model to generalize across various conditions. After preprocessing, images are annotated with labels.

The annotation provides ground truth data that the model will use to learn weed characteristics. Highquality annotations directly impact the model’s performance and are usually done by experts. In the model training phase, a deep learning model is trained on the annotated dataset. Popular architectures for image classification, such as CNNs, are often employed. During training, the model learns to distinguish weeds from crops by analyzing labeled examples and adjusting its internal parameters. Many hyperparameters are fine-tuned to balance training speed and accuracy, producing a model capable of identifying weed patterns accurately. The trained model undergoes validation, where it is tested on a separate validation dataset to evaluate its generalization performance. Key metrics like accuracy, precision, recall, and F1-score are calculated to assess the model’s ability to correctly classify weeds. Given an input image, the model classifies it and determines if a weed is present, identifying the weed type if applicable. The output is the classified weed type in the image, which can be displayed to end users, such as farmers or agricultural specialists. The workflow concludes with the “End” point, marking the completion of the weed classification process.

### 2.3 Model training

Various CNN-based models are deployed and trained on the images of cotton crop weeds. A CNN-based architecture consisting of several blocks with different numbers of convolutional layers with an increasing number of kernels in every block has been used for the weeds classification problem. The number of kernels in each filter varies from 32 kernels to 512 kernels till the last block of our proposed architecture. A number of key areas in the model’s architecture are tuned and optimized for improving the model detection and classification accuracy. Some of these key areas or techniques are listed below:

- Kernel Initializer
- Activation Function in every conv-layer
- Batch Normalization
- Max pooling
- Dropouts

An (f × f) filter convolves an (n × n) dimensional image. Convolution can be thought of simply as a dot product. The filter outputs an (n-f+1 × n-f+1) feature map after the convolution operation. Usually, the dimensions of the image are reduced when convolution happens at the edges of the image. An (f × f) filter acting on an (n × n) image has output dimensions (n - f + 1) × (n - f +1). Thus the image gets reduced in terms of dimensions after successive convolution operations and this affects the performance of the model. A common solution to this issue is zero padding. After each convolution operation, the boundaries of the image are padded with as many zeros as possible to maintain the original dimensions.

Kernel Initializer is a function used to initialize the initial weights (kernels of a filter in our case). Random initialization of the neural network weights results in more time to converge back to the global minima (minimum cost). For the initialization of weights, the HE-uniform kernel initializer is used to initialize the weights of kernels in every convolutional layer. It draws the initial weights from the truncated normal distribution, where *f_n_* is the number of input units.

An activation function is used in every convolutional layer to introduce the non-linearity to the summed weighted input and then feed it into the next layer. The activation function delays input to those neurons whose output is less effective by using a simple mathematical function. Some of the Activation functions used these days in neural networks are Sigmoid, Tanh, and Relu activation functions. But RelU is the most common activation function used in almost every Deep learning model Szandała (2021).

There are plenty of activation functions to use and ReLU is the common choice. ReLU function which is well known for its technique to handle the negative values such that it deactivates the neuron if the output of linear transformation is less than zero. It is far more effective than sigmoid and tanh activation functions and also computationally not as complex as other activation functions Rustam et al. (2022). CNNs are optimized because they reduce the number of trainable parameters. This helps the network fight the curse of dimensionality. The optimization in CNNs revolves around the fact that as the network gets deeper, very little information is required about specific locations of features. Time complexity is also reduced when reduction is done in dimensions and depth of data. For this reason, CNN takes less time than ANN on the same data Hasan et al. (2019).

Dimensions are reduced in two ways: Pooling layers are introduced after convolution layers to downsample the output feature maps. Pooling acts by keeping the important data in feature maps and discarding the less important ones hence reducing the dimensions. Pooling can be done in many ways for example Max Pooling and Average Pooling. A major goal in solving any machine learning problem is to make a model that generalizes well and is optimized. Optimization helps in getting the best possible results on the ‘training data’ while generalization is the measure of a model’s performance on unseen data. If optimization and generalization are not properly taken care of, then issues such as over-fitting and under-fitting arise.

Regularization is the process of regularizing or introducing some penalty term to the loss function when the model predicts. Regularization aims to reduce over-fitting. In dropout regularization, the dependency of the network on specific neurons is reduced and the model becomes more generalized and robust. The output from the pooling layer is fed to a regular neural network for further processing.

Hyperparameter tuning is a critical step in the design and optimization of deep learning models, especially in a complex application like weed identification, where model accuracy and robustness can significantly impact real-world results. In this study, we employed a systematic approach to tune the key hyperparameters, including learning rate, batch size, number of filters, dropout rate, and optimizer type. A grid search method was initially used to identify a range of values for each hyperparameter, based on prior studies and empirical testing. For the learning rate, values between 0.0001 and 0.01 were tested to balance convergence speed with stability. A batch size of 32 was selected after comparing values ranging from 16 to 128, balancing memory constraints with model performance. The dropout rate was optimized between 0.2 and 0.5 to reduce overfitting while maintaining generalization, with a final selection of 0.33 for dense layers based on validation performance. The model’s architecture used an increasing number of filters per convolutional layer, progressing from 32 to 512 filters, which was fine-tuned based on the complexity of the dataset. We used the Adam optimizer with default momentum settings after comparing performance with SGD and RMSprop, finding Adam provided more stable convergence. Each configuration was evaluated using k-fold cross-validation (with 10 folds) to mitigate overfitting and ensure robustness. Final hyperparameters were selected based on the model’s performance metrics, particularly validation accuracy and loss, as well as computational efficiency. This thorough tuning process ensured that the proposed model was optimized for both accuracy and computational feasibility, making it suitable for real-time agricultural applications.

Stride is a hyper-parameter and is defined as the number of steps n by which the pooling filter slides over the image. The pooling filter slides from left to right or down on the feature map and covers the whole feature map. The output from the pooling filter is termed the output channel and fed to the next convolution or ANN layer. Setting the stride hyper-parameter to n reduces the dimensions by n. The input image is fed into the convolutional layer of the model. The convolution operation is performed on every block such that a filter having n number of kernels of size (s × s) convolve with the input image having dimensions i × i traversing the whole image and learning some representation from the image. The output from the convolution layer is then passed to an activation function to introduce non-linearity and hence make the model capable of learning complex patterns.

Afterwards, batch normalization is applied which standardizes the activation output by introducing the batch normalization layer. Batch normalization reduces the number of epochs required to train the network and the complexity of the model. The output from the batch normalization is fed to the Max Pooling layer to fight over-fitting and reduce computational complexity by reducing the number of trainable parameters. After passing from a series of such blocks with increasing numbers of Kernels and such conv-layers the output from the base convolutional model fed into the dense-layer model after flatten them out.

A dense classifier, similar to the ones in regular ANNs, is connected to the convolution base. The output from the convolution base is flattened out since the dense layer expects single-dimensional input. The output layer provides the output in the form of probabilities for each distinct class. A soft-max activation function is used in the output layer to predict the output in the form of probabilities.

All comparison models (VGG-16, ResNet-101, DenseNet-121, and XceptionNet) were in fact refined by transfer learning on the particular weed dataset utilized for the proposed model in order to guarantee fairness. By fine-tuning these models, the comparisons become more justified and robust by matching them with the domain and data requirements of cotton weed categorization. Each model’s performance in this specific application was optimized through the use of transfer learning. The design of the suggested model, however, showed excellent performance even after fine-tuning, indicating its applicability for challenging, multiclass weed classification applications. **Table 1** shows the architectural comparison of various models used in this study.

**Table 1:** Comparison of model architectures.

Model	Pooling	Activation Function	Dropout Size	Filter Size
Proposed Optimized VGG	Max Pooling	ReLU, Softmax	0.2	3x3
VGG-16	Max Pooling	ReLU	0.5	3x3
ResNet101	Average Pooling	Softmax	Not used	1x1
DenseNet121	Average Pooling	ReLU	0.001	3x3
Xception	Average Pooling	ReLU	0.4	3x3

### 2.4 Model architecture

**Figure 4:**
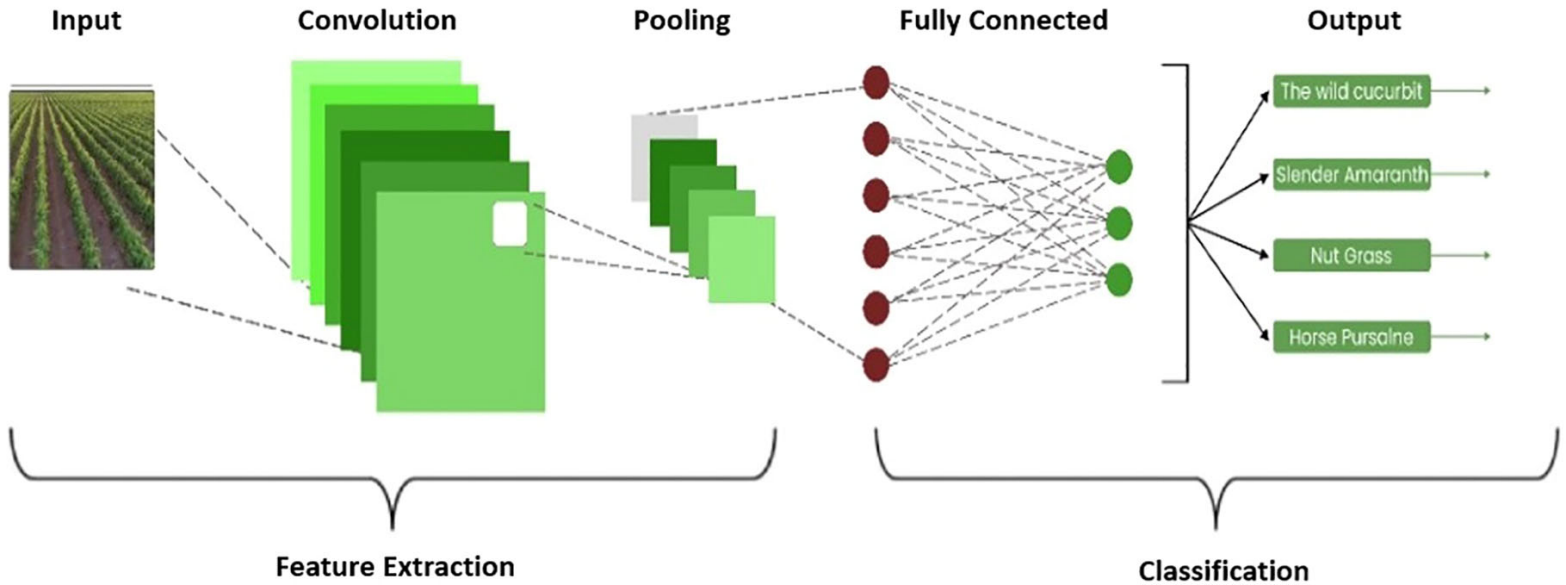
Architecture of the proposed model.

**Figure 4** shows the architecture of the proposed CNN model for cotton-based weed classification. The proposed model contains several convolutions, pooling, fully connected, and drop-out layers whose details are provided here.

1. Convolutional Layer
  • Conv2D filters: 32, 64, 128, 256, 512
  • Conv2D kernel size: (3, 3) for the first two Conv2D layers, (5, 5) for the next two, and (7, 7) for the last three
  • Activation function: ReLU
  • Kernel initializer: He uniform
  • Padding: ‘SAME’ for all Conv2D layers
  • Kernel regularizer: L2 regularization with a coefficient of 0.001 for all Conv2D layers
2. Pooling Layers
  • MaxPooling2D with a pool size of (2, 2) after each pair of Conv2D layers
3. Batch Normalization
  • Applied after every pair of Conv2D layers
4. Dropout
  • Applied after the second and fourth pairs of Conv2D layers, and after the Dense layer
  • Dropout rates: 0.2 for Conv2D layers and 0.33 for the Dense layer
5. Dense Layers
  • Dense layer with 256 units and ReLU activation
  • Dense output layer with 4 units and softmax activation (assuming it’s a classification task with 4 classes)

### 2.5 Hyper parameters working

1. Convolutional Layers
  • Filters: The number of filters progressively increases from 32 to 512 across the convolutional layers, allowing the model to capture increasingly complex features.
  • Kernel Size: The kernel size varies from (3, 3) to (7, 7) across layers, enabling the network to capture features at different scales.
  • Activation Function: ReLU activation function is used to introduce non-linearity into the model.
  • Kernel Initializer: He uniform initialization method is employed, which initializes weights in a way that is more suitable for ReLU activations, aiding in faster convergence.
  • Padding: ‘SAME’ padding is utilized to ensure that the spatial dimensions of the input and output feature maps remain the same.
  • Kernel Regularizer: L2 regularization with a coefficient of 0.001 is applied to all convolutional layers to prevent overfitting and promote generalization.
2. Pooling Layers
  • MaxPooling2D: Applied with a pool size of (2, 2) after each pair of convolutional layers, reducing the spatial dimensions of the feature maps while retaining important information.
3. Batch Normalization
  • Batch normalization is applied after every pair of convolutional layers, helping to stabilize and accelerate the training process by normalizing the activations.
4. Dropout
  • Dropout regularization is applied after the second and fourth pairs of convolutional layers, as well as after the dense layer. Dropout rates of 0.2 are used for convolutional layers, and 0.33 for the dense layer, respectively, to prevent overfitting by randomly dropping a proportion of neurons during training.
5. Dense Layers
  • Dense Layer 1: Consists of 256 units with ReLU activation, providing a high- capacity representation of the extracted features.
  • Dense Output Layer: Comprises 4 units with softmax activation, suitable for multi-class classification tasks with 4 classes, producing probability distributions over the classes.

## 3 Experiments and results

In this section, all the details of the experiments and results based on the performance metrics for the proposed CNN model are discussed.

### 3.1 Experimental setup

This study performs experiments using Google Colab on an Intel Core i7 system with 16GB RAM. Python is used to implement the selected CNN-based models. A number of same labeled images, which were 14,000 in total were used and the dataset was divided into 80% to 20%, for training and testing, respectively. **Table 2** provides the details for class-wise train-test split for experiments.

**Table 2:** Class-wise samples for training and testing.

Class	Training	Testing	Total
Wild Cucurbit	1,840	460	2,300
Slender Amaranth	1,840	460	2,300
Nut Grass	1,840	460	2,300
Horse Purslane	1,840	460	2,300
Common Puncture Vine	1,840	460	2,300
Trefoil	1,920	480	2,400

### 3.2 Performance metrics

In the performance metrics, the two most common parameters are used i.e. accuracy and loss. This whole process was accomplished through the confusion table. Accuracy provides a summary of the performance of the model and in often cases is not enough to decide if the model is satisfactory or not Asad and Bais (2020). Accuracy is calculated using the following.


(1) $$Accuracy=\frac{CP}{TP}$$

where *CP* corresponds to the number of correct predictions and *TP* corresponds to the total predictions. The results seem good with high validation accuracy and low validation loss in predicting the weeds classification by the proposed model. Loss is calculated using a loss function. In the proposed model, categorical cross-entropy is used as the loss function to find the loss score Xu and Chang (2017).


(2) $$loss=-\sum_{i}^{totaloutputs}y_{i}.log{\hat{y}}_{i}$$

In addition to accuracy and loss, we evaluated the model’s performance using Precision, Recall, and F1 scores. These metrics provide a more nuanced understanding of the model’s ability to accurately classify different weed types, addressing not only overall accuracy but also the model’s precision in identifying positive instances (Precision), its sensitivity to true positive cases (Recall), and the balance between the two (F1 Score). Precision, Recall, and F1 scores are calculated as follows:


(3) $$Precision=\frac{TP}{TP+FP}$$

(4) $$Precision=\frac{TP}{TP+FN}$$

(5) $$Precision=2\times \frac{Precision\times Recall}{Precison+Recall}$$

The proposed model achieved high scores across these metrics, with Precision, Recall, and F1 scores consistently above 0.98, indicating its reliability in correctly classifying various weed species. These metrics are particularly valuable for understanding the model’s performance under conditions of class imbalance or in scenarios where false positives or false negatives carry different consequences, as often encountered in agricultural applications.

The ground truth and CNN score for each class *i* in the total number of classes are $y_{i}$ and ${\hat{y}}_{i}$ , respectively. Before computing the Loss, an activation function (Sigmoid/Softmax) is applied to the scores. After finding out the loss score the next target is to reduce the error score by using an optimizer (convex optimization) and update weights of models in every epoch.

### 3.3 Experimental results

**Figure 5:**
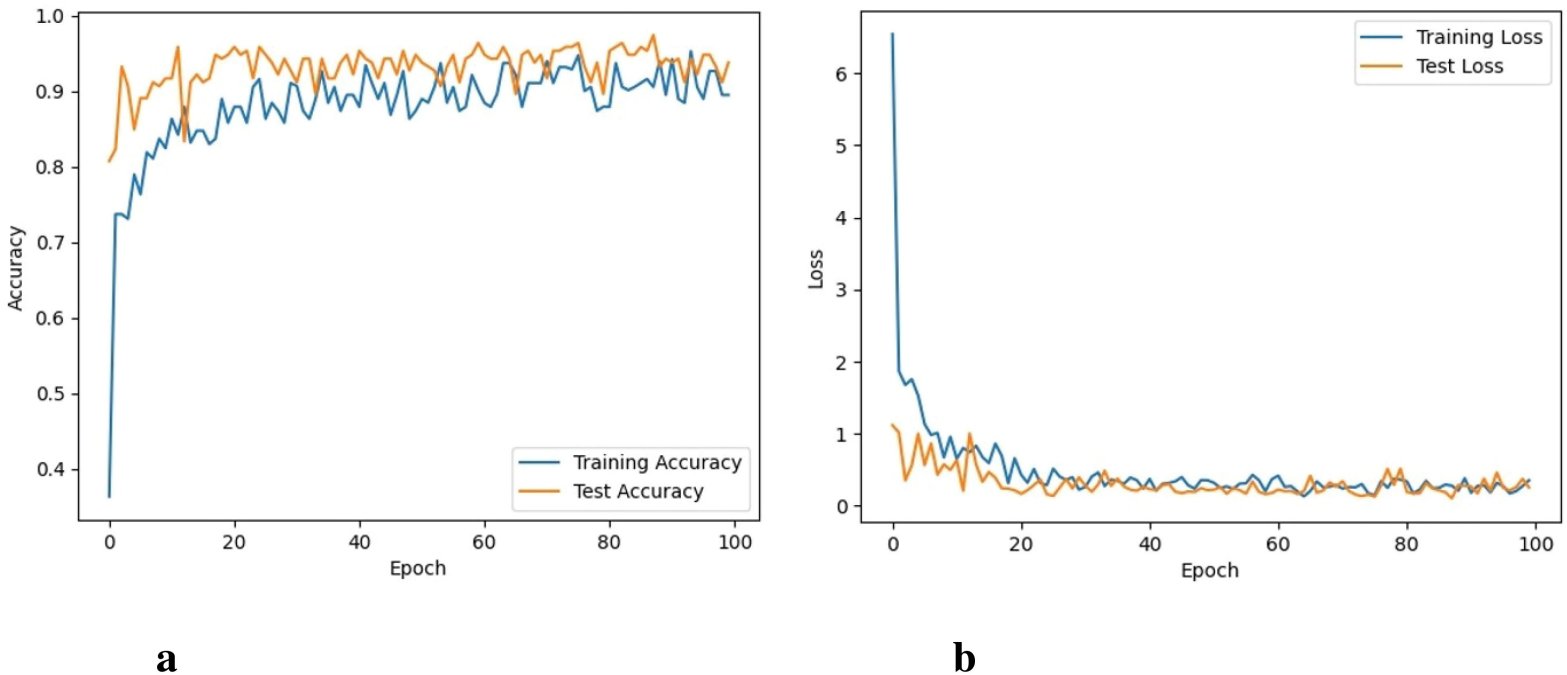
Accuracy and loss of the proposed approach, **(A)** Training and testing accuracy, and **(B)** Training and testing loss.

The model is trained for 100 epochs. The classification metrics are calculated after the 100 epochs. The proposed model took almost 32 hours to perform 100 iterations of training. The graphs for training accuracy, training loss, validation accuracy, and validation loss of the classification of 6 different types of weeds are given here. The training accuracy of the proposed model was 98.3% with a training loss of 0.041 as shown in **Figures 5A, B**.

**Figure 6:**
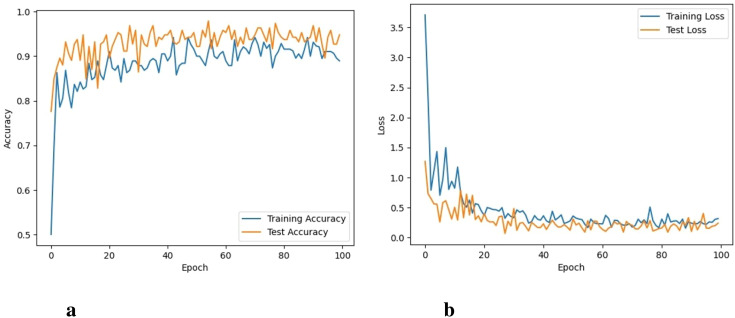
Accuracy and loss of the VGG16 model, **(A)** Training and testing accuracy, and **(B)** Training and testing loss.

It took 33 hours for VGG-16 to perform 100 iterations. VGG-16 was able to reach a training accuracy of 94.3% with a training loss of 0.062. Its graphs can be seen in **Figures 6A, B**. Compared to the training and testing loss of the proposed CNN model, the difference between the training and testing loss of the VGG model is higher. In addition, it shows lower accuracy compared to the proposed model.

**Figure 7:**
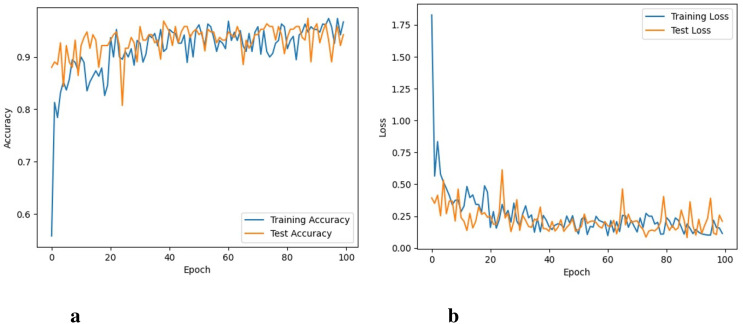
Accuracy and loss of the ResNet101 model, **(A)** Training and testing accuracy, and **(B)** Training and testing loss.

The ResNet101 took 44 hours to perform 100 iterations. ResNet-101 was able to obtain a training accuracy of 96.1% and a training loss of 0.043. **Figures 7A, B** depicts graphs for training, validation accuracy, and training as well as validation losses. The ResNet-101 model shows better results than the VGG16 model, however, its performance is not as good as shown by the proposed model.

**Figure 8:**
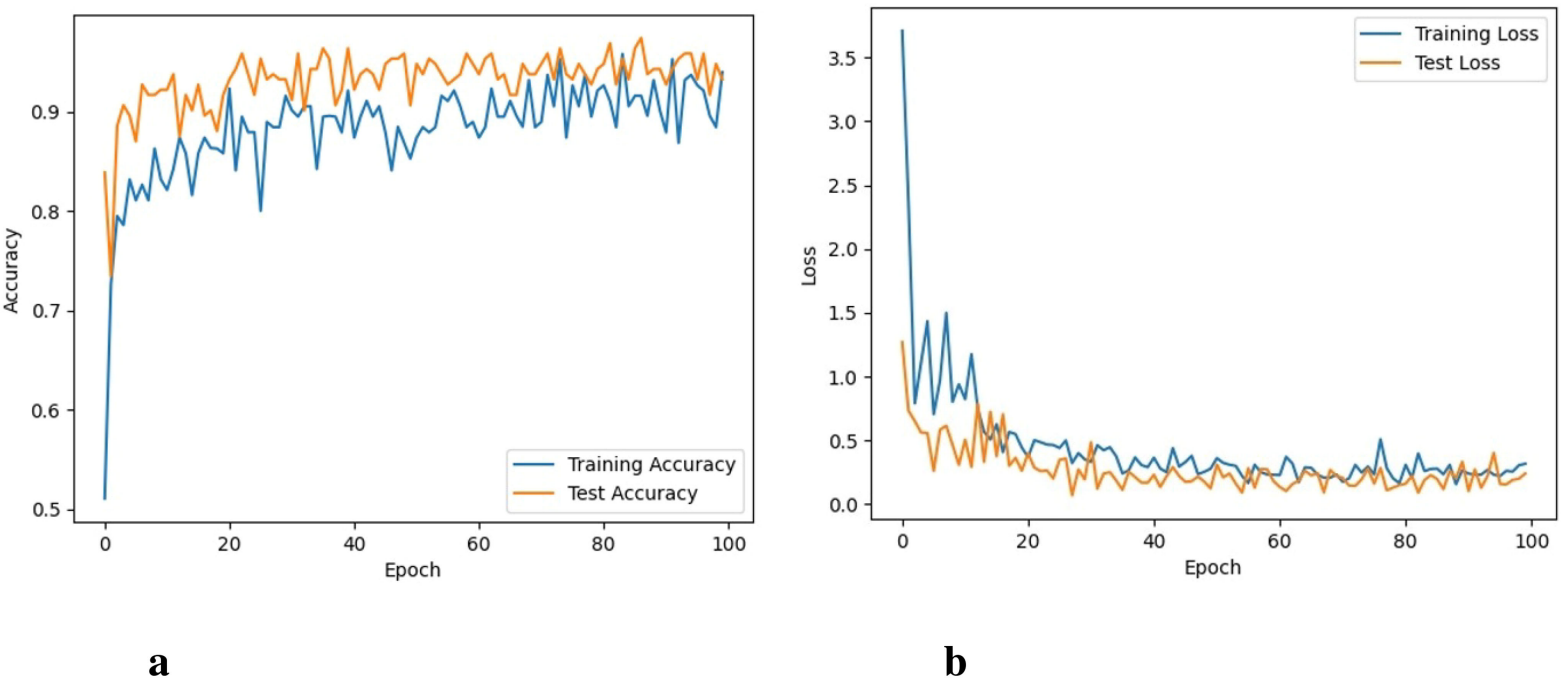
Accuracy and loss of the DenseNet model, **(A)** Training and testing accuracy, and **(B)** Training and testing loss.

It took 48 hours for DenseNet-121 to perform 100 iterations. **Figures 8A, B** depicts the graphs for both training and validation accuracy and training and validation loss. It can be seen that training accuracy reaches up to 96.4% and training loss goes to 0.045. The performance of the DenseNet-121 and ResNet101 is almost similar.

**Figure 9:**
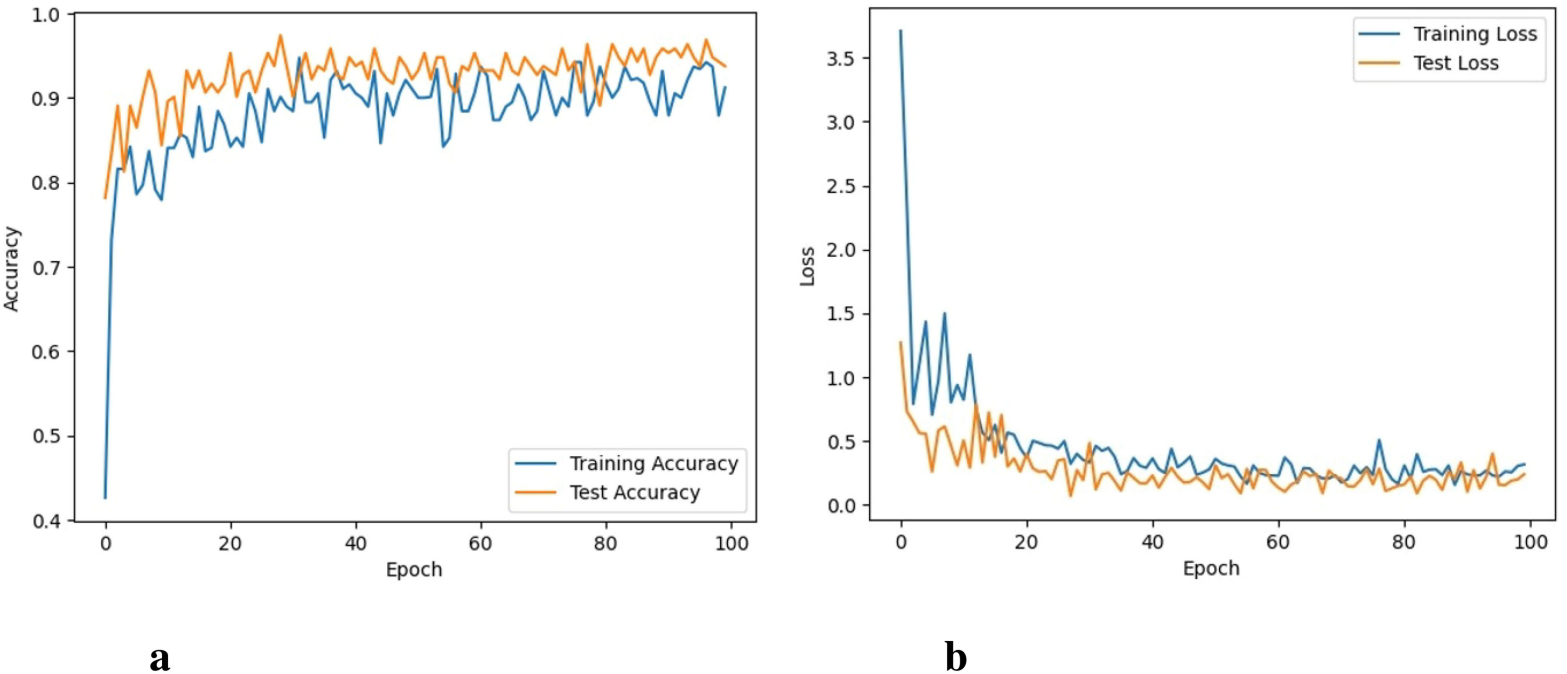
Accuracy and loss of the Xception model, **(A)** Training and testing accuracy, and **(B)** Training and testing loss.

It took 18 hours for Xception to perform 100 iterations. Graphs for training accuracy, validation accuracy, training loss, and validation loss are shown in **Figures 9A, B**. Training accuracy reaches up to 95.2% and training loss goes to 0.056.

**Figure 10:**
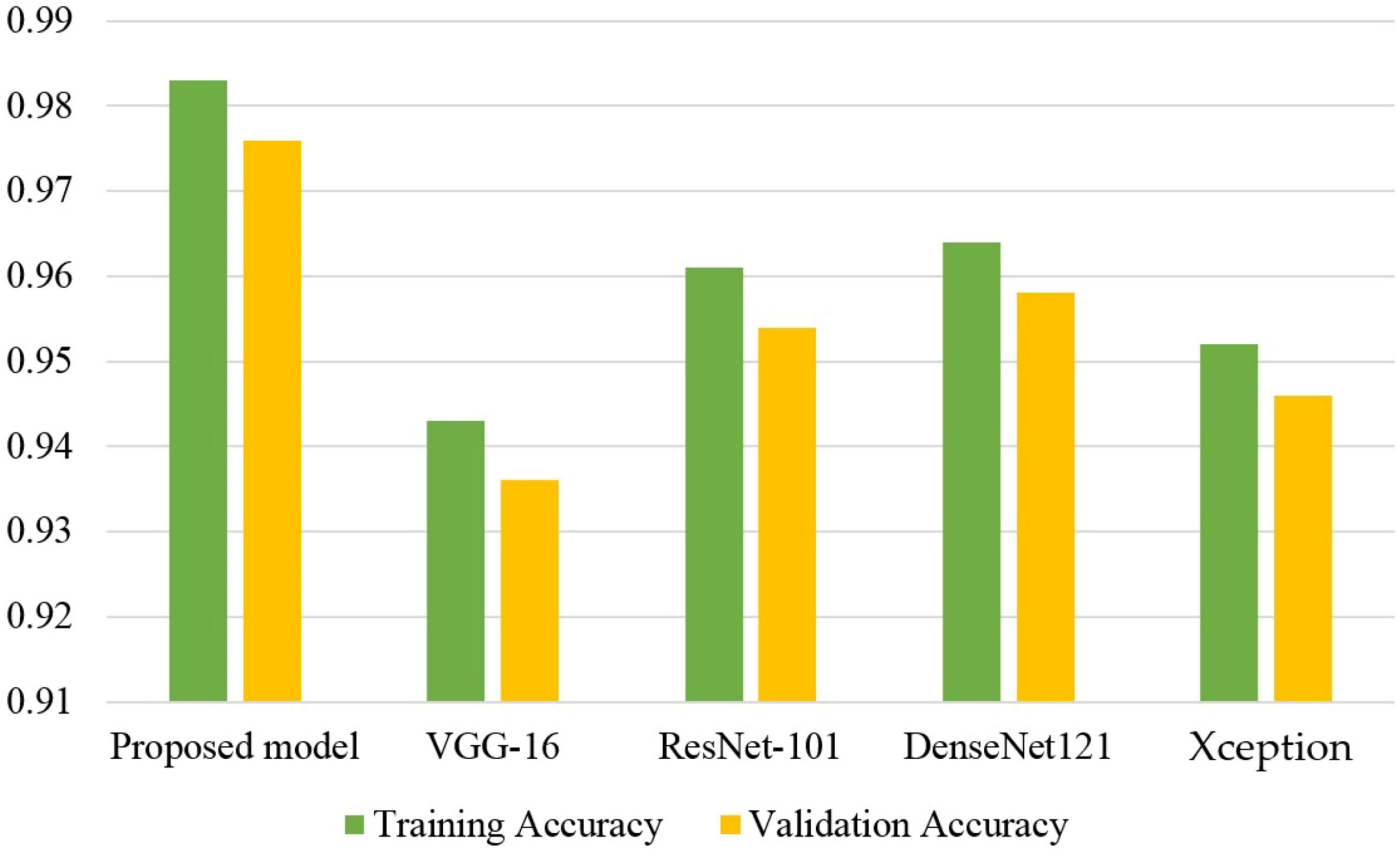
Accuracy comparison for all models.

Comparison graphs for the accuracy of all models are presented in **Figure 10**. The results indicate that the proposed model performs much better than other CNN models in terms of training and testing accuracy. The VGG16 model shows the poorest results compared to other models while the performance of ResNet-101 and DenseNet121 models show marginally different performance.

**Figure 11:**
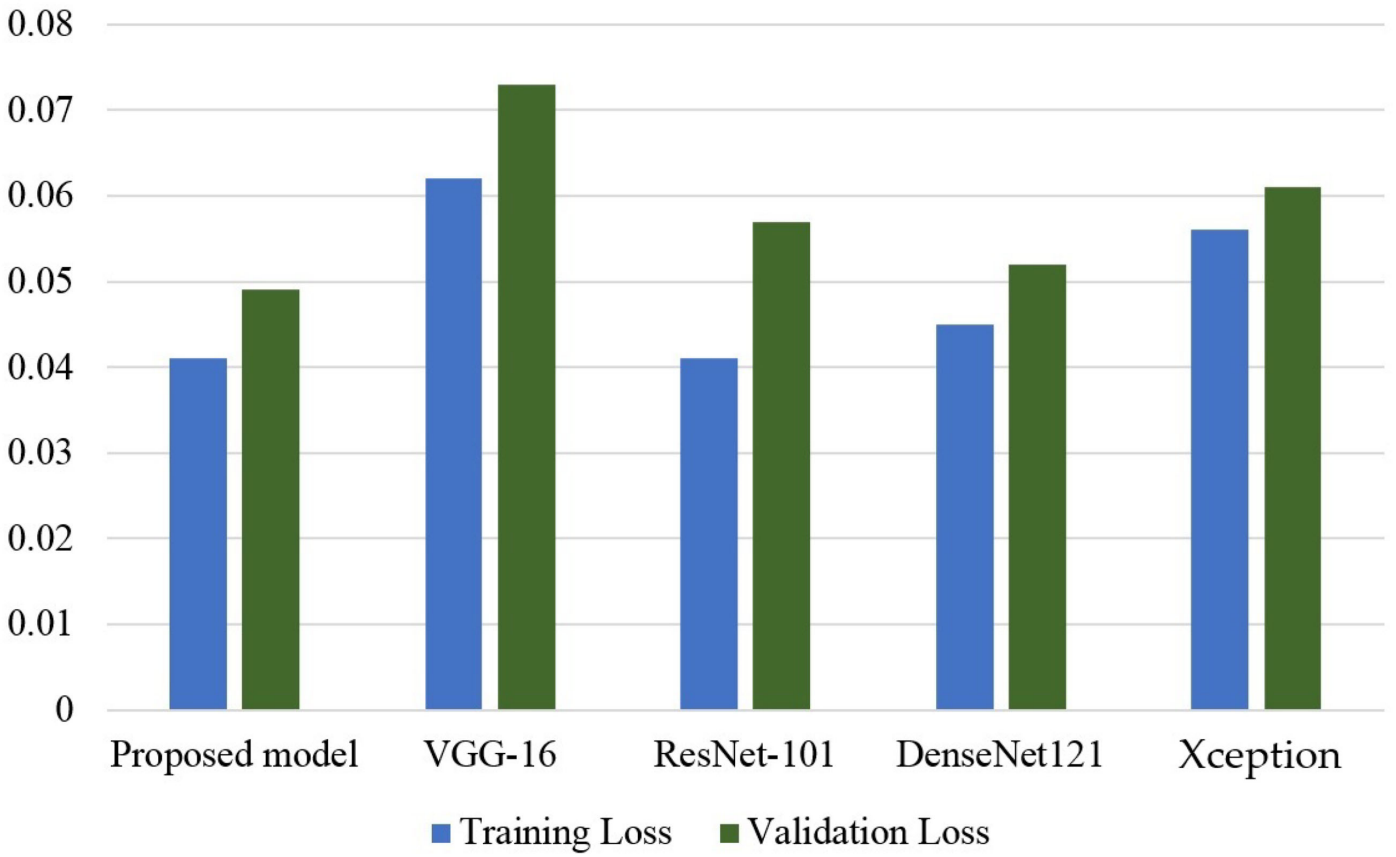
Loss comparison of all models.

**Figure 11** shows the results of all CNN models in terms of training and testing loss. The VGG-16 model observes the highest training and testing loss, followed by the Xception model. Although the ResNet-101 model shows a very low training and testing loss, it is marginally higher than what is obtained by the proposed CNN model.

To evaluate the results of the proposed model, results are compared with different CNN models on the same dataset and comparative results are shown in **Table 3**. CNN models were used for six different types of weed classification. All CNN models were tested in the specified environment, in which the result of the proposed model was better than the rest of the models.

**Table 3:** Performance comparison of various CNN models.

Models	Training Accuracy	Training Loss	Validation Accuracy	Validation Loss
Proposed	0.983	0.041	0.976	0.049
VGG-16	0.943	0.062	0.936	0.073
ResNet-101	0.961	0.043	0.954	0.057
DenseNet-121	0.964	0.045	0.958	0.052
Xception	0.952	0.056	0.946	0.061

In the proposed model, dropout regularized CNN-based architecture is used for the classification of weeds. The results of the proposed architecture, as shown in **Table 4**, indicate a superior performance of the proposed model compared to the other four well-known CNN models. The table shows accuracy and loss values for the proposed model and various state-of-the-art architectures trained via transfer learning. The accuracy of VGG-16 is 94.3%, ResNet-101 is 96.1%, DenseNet-121 is 96.4% and for Xception, the accuracy is 95.2%. The proposed model proves to be better with the resulting detection and classification accuracy of 98.3% than other models.

**Table 4:** Performance comparison of all models concerning precision, recall, etc.

Models	Accuracy	Precision	Recall	F1 score
Proposed	0.983	0.9862	0.9861	0.9818
VGG-16	0.943	0.9418	0.9404	0.9412
ResNet-101	0.961	0.9661	0.9671	0.9623
DenseNet-121	0.964	0.9632	0.9711	0.9636
Xception	0.952	0.9497	0.9496	0.9510

Results were changed on the basis of two different reasons as the datasets were collected in different environmental conditions like early morning (05:50 am to 6:20 am), morning (06:40 am to 09:00 am), noon (12:00 pm to 01:00 pm), afternoon (03:00 pm to 04:00 pm) and before sunset (05:00 pm to 06:00 pm). Parameters were changed of the proposed model against the existing models.

**Figure 12:**
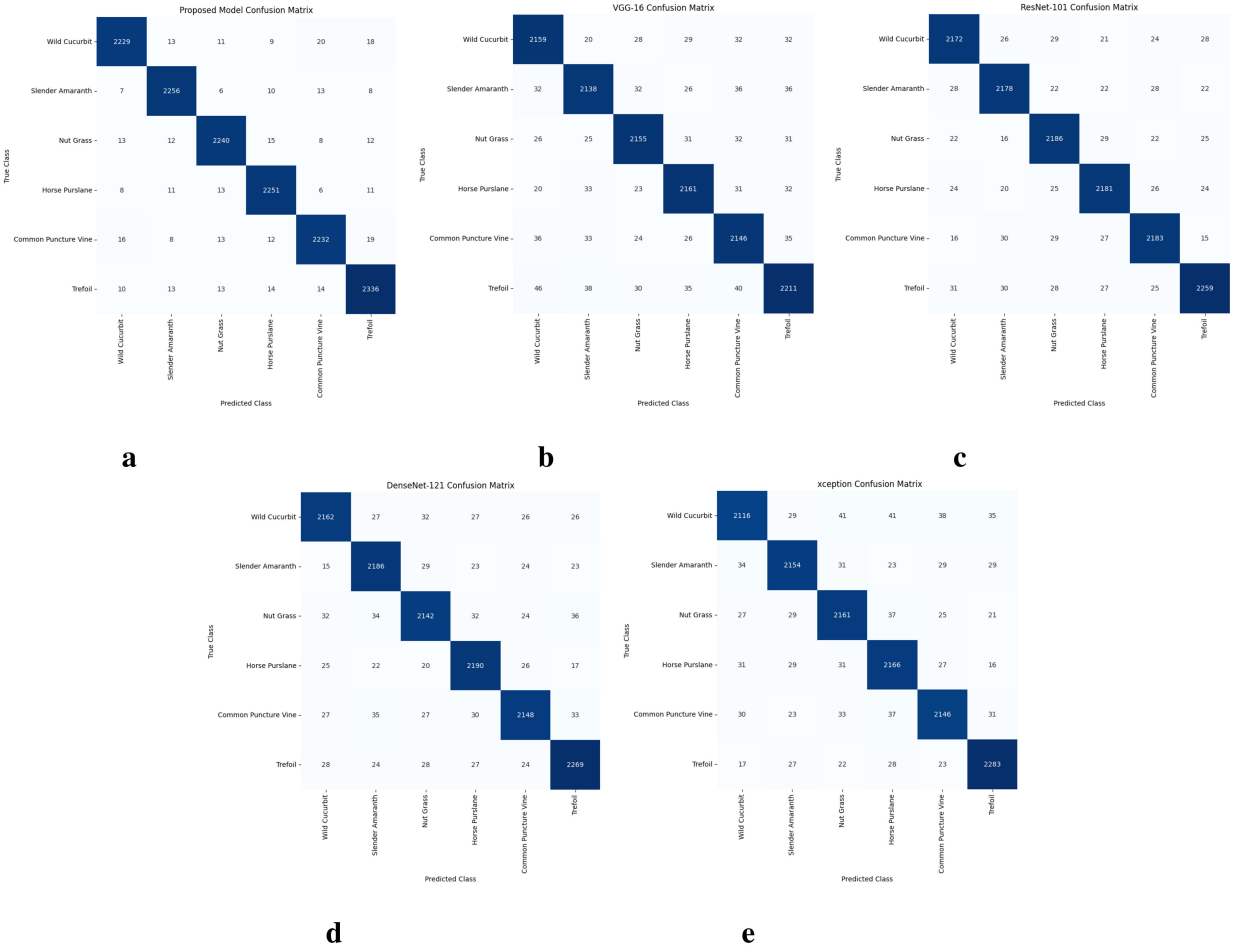
Confusion matrices for all models, **(A)** Proposed model, **(B)** VGG-16 model, **(C)** ResNet-101 model, **(D)** DenseNet-121 model, and **(E)** Xception model.

In addition to accuracy, other performance metrics like F1 score, precision, etc. are better compared to other CNN models. For example, proposed models 0.9862, 0.9861, and 0.9818 scores for precision, recall, and F1 score, respectively are much better than ResNet-101 and DenseNet-121 which performed really well. Moreover, performance concerning the number of correct predictions (CP) and wrong predictions (WP) is also illustrated in **Figure 12**. The lowest number of CP is recorded with VGG-16 which is 12,970 out of a total of 13,900, and ultimately it has the highest number of WP of 930. The Xception model performs better than VGG-16 and predicts 13,026 samples correctly. DenseNet-121 and ResNet-101 perform better concerning correct predictions and make 13,097 and 13,159 predictions correctly. The proposed model performs the best with 13,544 correct predictions and only 356 predictions are wrong.

### 3.4 K-fold cross-validation

To evaluate the model’s performance concerning robustness and mitigate the risk of overfitting, k-fold cross-validation with 10 folds is employed. This involves splitting the dataset into 10 folds, training the model on 9 folds, and validating it on the remaining one fold. This process is repeated 10 times, ensuring that each fold serves as both a training and validation set. The final performance metrics are typically computed as the average across all folds, providing a more reliable estimate of the model’s generalization performance. By incorporating k-fold cross-validation, the proposed CNN model aims to prevent overfitting and generalize well to unseen data in the context of classification tasks. Results given in **Table 5** indicate superior performance of the proposed model in all folds concerning training and testing accuracy, thereby proving its robustness.

**Table 5:** K-fold cross-validation results.

K-Fold	Training Accuracy	Training Loss	Validation Accuracy	Validation Loss
1	0.95	0.05	0.94	0.06
2	0.95	0.05	0.97	0.03
3	0.96	0.04	0.92	0.08
4	0.95	0.05	0.89	0.11
5	0.97	0.03	0.97	0.03
6	0.93	0.07	0.92	0.08
7	0.93	0.07	0.92	0.08
8	0.93	0.07	0.92	0.08
9	0.97	0.03	0.96	0.04
10	0.93	0.07	0.96	0.04
Average	0.947	0.053	0.937	0.063

### 3.5 Comparison with existing models

Further investigation has been conducted to provide an in-depth comparison of the proposed weed classification approach against well-established models from the existing literature. Several studies have shown promising results in weed classification, with machine learning and deep learning techniques achieving remarkable accuracy levels across various datasets. For instance, research works such as Vypirailenko et al. (2021); Dataset Weed Images (2022); Asad and Bais (2020); Grace et al. (2021) present stateof-the-art performances, illustrating the strengths of these methods in controlled settings. Specifically, Dataset Weed Images (2022) and Asad and Bais (2020) achieve classification accuracies of 98.8% and 98.23%, respectively, showcasing highly effective models that are finely tuned for binary or limited-class weed identification tasks. Likewise, Benos et al. (2021) adopts an SVM-based technique, obtaining a 96.70% accuracy rate, which emphasizes the continued relevance of traditional machine learning models for specific weed classification scenarios where data variability is limited.

However, many of these models encounter limitations when applied to multiclass weed classification, which requires distinguishing between a larger number of weed types that may exhibit subtle visual differences. These models often struggle with scalability and generalization in the face of increased complexity and inter-class similarities, which can lead to misclassification or reduced accuracy. The proposed approach, by contrast, is specifically optimized to handle multiclass classification by leveraging an enhanced feature extraction process that captures detailed and distinguishing features across a wide range of weed species. This ability to discern fine-grained differences allows the model to maintain high accuracy across diverse weed types, thereby addressing the scalability challenges seen in other methods.

Moreover, the proposed model incorporates techniques such as adaptive pooling layers and optimized convolutional kernels that are fine-tuned to balance precision and computational efficiency, making it more suitable for deployment in real-time agricultural settings. **Table 6** provides a detailed performance comparison, indicating that our approach consistently outperforms existing models in multiclass classification tasks. Not only does our method yield higher accuracy, but it also demonstrates robustness against variations in lighting, angle, and occlusion, conditions that are common in real-world environments but often underrepresented in controlled experimental setups. This robustness makes the proposed model particularly valuable for practical applications in precision agriculture, where accurate weed identification is crucial for targeted herbicide application and resource management.

**Table 6:** Comparison of performance with existing studies.

Reference	Classification	Model	Accuracy
Vypirailenko et al. (2021)	Multiclass	ResNet	93.45% accuracy
Dataset Weed Images (2022)	Binary class	YOLOv3	98.8%
Asad and Bais (2020)	Binary class	DNN	98.23%
Grace et al. (2021)	Binary class	CNN	89% accuracy
Jin et al. (2021)	Multiclass	CenterNet	95.3% accuracy
Olsen et al. (2019)	Binary class	ResNet50	97.6%
Knoll et al. (2019)	Multiclass	CNN	96.82%
Benos et al. (2021)	Multiclass	SVM	96.70%
Proposed	Multiclass	CNN	98.30%

The proposed model is especially tailored for the particular environmental and visual challenges of weed identification in cotton fields, even if it only marginally outperforms well-known architectures in terms of accuracy (98.3% vs. ResNet-101’s 97.1%). To manage fine-grained visual distinctions, it uses special features like adaptive pooling and customized convolutional filters. Compared to generalized architectures, this focus on domain-specific optimizations makes it more feasible for precision agriculture and more dependable in real-world situations where weed species exhibit small visual variations.

## 4 Conclusions

Weeds are dangerous and destructive to various crops including cotton. Weeds have the potential to destroy cotton crops resulting in huge economic losses. Previously, there were various methods based on computer vision for weed classification and the research field is still active and undergoing further research. For the detection and classification of weeds in cotton crops, an improved approach based on a dropout regularized CNN model has been proposed. The proposed work illustrates an improved methodology for the classification of weeds in cotton plants. The model is rigorously investigated through experiments, crossvalidation, and performance comparison with the already available state-of-the-art models. Experimental results indicate superior performance of the proposed model over other approaches. The proposed work also forms the basis for developing various applications in the field of agriculture and farming. The applications of this research will help the farmers to obtain higher yields by detecting the weeds in their farms. In the future, robotic-based solutions will be made for weed identification, classification, and spraying of weedicides.

## Data Availability

The raw data supporting the conclusions of this article will be made available by the authors, without undue reservation.

## References

[B1] Abouzahir S. Sadik M. Sabir E. (2018). “Enhanced approach for weeds species detection using machine vision,” in 2018 International Conference on Electronics, Control, Optimization and Computer Science (ICECOCS) (IEEE). New York City, U.S: IEEE 1–6. doi: 10.1109/ICECOCS.2018.8610505

[B2] Ahmad A. Saraswat D. Aggarwal V. Etienne A. Hancock B. (2021). Performance of deep learning models for classifying and detecting common weeds in corn and soybean production systems. Comput. Electron. Agric. 184, 106081. doi: 10.1016/j.compag.2021.106081

[B3] Asad M. H. Bais A. (2020). Weed detection in canola fields using maximum likelihood classification and deep convolutional neural network. Inf. Process. Agric. 7, 535–545. doi: 10.1016/j.inpa.2019.12.002

[B4] Benos L. Tagarakis A. C. Dolias G. Berruto R. Kateris D. Bochtis D. (2021). Machine learning in agriculture: A comprehensive updated review. Sensors 21, 3758. doi: 10.3390/s21113758 34071553 PMC8198852

[B5] Capinera J. L. (2005). Relationships between insect pests and weeds: an evolutionary perspective. Weed Sci. 53, 892–901. doi: 10.1614/WS-04-049R.1

[B6] Dadashzadeh M. Abbaspour-Gilandeh Y. Mesri-Gundoshmian T. Sabzi S. Hernández-Hernández J. L. Hernández-Hernández M.. (2020). Weed classification for site-specific weed management using an automated stereo computer-vision machine-learning system in rice fields. Plants 9, 559. doi: 10.3390/plants9050559 32349459 PMC7284472

[B7] Dataset Weed Images (2022). Weed images: The source for images of weeds and weed management in agriculture. Available online at: https://www.weedimages.org/ (Accessed November 20, 2024).

[B8] Dokic K. Blaskovic L. Mandusic D. (2020). “From machine learning to deep learning in agriculture– the quantitative review of trends,” in IOP Conference Series: Earth and Environmental Science, Bristol, England vol. 614. (Bristol, England: IOP Publishing), 012138.

[B9] Escalante H. Rodríguez-Sánchez S. Jiménez-Lizárraga M. Morales-Reyes A. de la Calleja J. Vazquez R. (2019). Barley yield and fertilization analysis from uav imagery: a deep learning approach. Int. J. Remote Sens. 40, 2493–2516. doi: 10.1080/01431161.2019.1577571

[B10] Espinoza M. A. M. (2020). Using Machine Learning For Weed Identification And Yield Prediction Of Strawberries. Ph.D. thesis (Pomona: California State Polytechnic University).

[B11] Grace R. K. Anitja J. Sivaranakrishnan R Sivakumari M. S. S. (2021). Crop and weed classification using deep learning. Turkish J. Comput. Mathematics Educ. (TURCOMAT) 12, 935–938.

[B12] Grigorescu S. Trasnea B. Cocias T. Macesanu G. (2020). A survey of deep learning techniques for autonomous driving. J. Field Robotics 37, 362–386. doi: 10.1002/rob.21918

[B13] Guo Y. Zhang J. Yin C. Hu X. Zou Y. Xue Z.. (2020). Plant disease identification based on deep learning algorithm in smart farming. Discrete Dynamics Nat. Soc. 2020, 1–11. doi: 10.1155/2020/2479172

[B14] Hasan M. Ullah S. Khan M. J. Khurshid K. (2019). Comparative analysis of svm, ann and cnn for classifying vegetation species using hyperspectral thermal infrared data. Int. Arch. Photogrammetry Remote Sens. Spatial Inf. Sci. 42, 1861–1868. doi: 10.5194/isprs-archives-XLII-2-W13-1861-2019

[B15] Imran M. A. Ali A. Ashfaq M. Hassan S. Culas R. Ma C. (2018). Impact of climate smart agriculture (csa) practices on cotton production and livelihood of farmers in punjab, Pakistan. Sustainability 10, 2101. doi: 10.3390/su10062101

[B16] Jayaraman P. P. Yavari A. Georgakopoulos D. Morshed A. Zaslavsky A. (2016). Internet of things platform for smart farming: Experiences and lessons learnt. Sensors 16, 1884. doi: 10.3390/s16111884 27834862 PMC5134543

[B17] Jin X. Che J. Chen Y. (2021). Weed identification using deep learning and image processing in vegetable plantation. IEEE Access 9, 10940–10950. doi: 10.1109/Access.6287639

[B18] Khan S. Tufail M. Khan M. T. Khan Z. A. Anwer S. (2021). Deep-learning-based spraying area recognition system forunmanned-aerial-vehicle-based sprayers. Turkish J. Electrical Eng. Comput. Sci. 29, 241–256. doi: 10.3906/elk-2004-4

[B19] Knoll F. J. Czymmek V. Harders L. O. Hussmann S. (2019). Real-time classification of weeds in organic carrot production using deep learning algorithms. Comput. Electron. Agric. 167, 105097. doi: 10.1016/j.compag.2019.105097

[B20] Kumar S. Bhowmick M. K. Ray P. (2021). Weeds as alternate and alternative hosts of crop pests. Indian Journal of Weed Science 53 (1), 14–29. doi: 10.5958/0974-8164.2021.00002.2

[B21] Luo T. Zhao J. Gu Y. Zhang S. Qiao X. Tian W.. (2023). Classification of weed seeds based on visual images and deep learning. Inf. Process. Agric. 10, 40–51. doi: 10.1016/j.inpa.2021.10.002

[B22] Olsen A. Konovalov D. A. Philippa B. Ridd P. Wood J. C. Johns J.. (2019). Deepweeds: A multiclass weed species image dataset for deep learning. Sci. Rep. 9, 2058. doi: 10.1038/s41598-018-38343-3 30765729 PMC6375952

[B23] Ruslan R. Khairunniza-Bejo S. Jahari M. Ibrahim M. F. (2022). Weedy rice classification using image processing and a machine learning approach. Agriculture 12, 645. doi: 10.3390/agriculture12050645

[B24] Rustam F. Ishaq A. Munir K. Almutairi M. Aslam N. Ashraf I. (2022). Incorporating cnn features for optimizing performance of ensemble classifier for cardiovascular disease prediction. Diagnostics 12, 1474. doi: 10.3390/diagnostics12061474 35741283 PMC9221641

[B25] Saiz-Rubio V. Rovira-Más F. (2020). From smart farming towards agriculture 5.0: A review on crop data management. Agronomy 10, 207. doi: 10.3390/agronomy10020207

[B26] Szandała T. (2021). Review and comparison of commonly used activation functions for deep neural networks. Bio-inspired Neurocomputing 903, 203–224. doi: 10.1007/978-981-15-5495-7_11

[B27] Valicharla S. K. (2021). Weed recognition in agriculture: A mask r-cnn approach. doi: 10.33915/etd.8102

[B28] Vypirailenko D. Kiseleva E. Shadrin D. Pukalchik M. (2021). “Deep learning techniques for enhancement of weeds growth classification,” in 2021 IEEE International Instrumentation and Measurement Technology Conference (I2MTC) (Bristol, England: IEEE), 1–6.

[B29] Xu Z. Chang L. (2017). Identification and Control of Common Weeds Vol. 3 (Singapore: Springer). doi: 10.1007/978-981-10-5403-7

[B30] Yu J. Sharpe S. M. Schumann A. W. Boyd N. S. (2019). Deep learning for image-based weed detection in turfgrass. Eur. J. Agron. 104, 78–84. doi: 10.1016/j.eja.2019.01.004

